# Epidemiology of recreational exposure to freshwater cyanobacteria – an international prospective cohort study

**DOI:** 10.1186/1471-2458-6-93

**Published:** 2006-04-11

**Authors:** Ian Stewart, Penelope M Webb, Philip J Schluter, Lora E Fleming, John W Burns, Miroslav Gantar, Lorraine C Backer, Glen R Shaw

**Affiliations:** 1 National Research Centre for Environmental Toxicology, University of Queensland, 39 Kessels Road, Coopers Plains, QLD 4108, Australia; 2 School of Population Health, University of Queensland, Herston Road, Herston, QLD 4006, Australia; 3 Cooperative Research Centre for Water Quality and Treatment, PMB 3, Salisbury, SA 5108, Australia; 4 Queensland Institute of Medical Research, 300 Herston Road, Herston, QLD 4006, Australia; 5 Faculty of Health and Environmental Sciences, Auckland University of Technology, Private Bag 92006, Auckland 1020, New Zealand; 6 NIEHS Marine & Freshwater Biomedical Sciences Center, University of Miami, FL 33149, USA; 7 PBS&J, 701 San Marco Blvd., Suite 1201, Jacksonville, FL 32207–8175, USA; 8 Department of Biology, Florida International University, Miami, FL 33199, USA; 9 National Center for Environmental Health, Centers for Disease Control and Prevention, Atlanta, GA 30333, USA; 10 School of Public Health, Griffith University, University Drive, Meadowbrook, QLD 4131, Australia

## Abstract

**Background:**

Case studies and anecdotal reports have documented a range of acute illnesses associated with exposure to cyanobacteria and their toxins in recreational waters. The epidemiological data to date are limited; we sought to improve on the design of some previously conducted studies in order to facilitate revision and refinement of guidelines for exposure to cyanobacteria in recreational waters.

**Methods:**

A prospective cohort study was conducted to investigate the incidence of acute symptoms in individuals exposed, through recreational activities, to low (cell surface area <2.4 mm^2^/mL), medium (2.4–12.0 mm^2^/mL) and high (>12.0 mm^2^/mL) levels of cyanobacteria in lakes and rivers in southeast Queensland, the central coast area of New South Wales, and northeast and central Florida. Multivariable logistic regression analyses were employed; models adjusted for region, age, smoking, prior history of asthma, hay fever or skin disease (eczema or dermatitis) and clustering by household.

**Results:**

Of individuals approached, 3,595 met the eligibility criteria, 3,193 (89%) agreed to participate and 1,331 (37%) completed both the questionnaire and follow-up interview. Respiratory symptoms were 2.1 (95%CI: 1.1–4.0) times more likely to be reported by subjects exposed to high levels of cyanobacteria than by those exposed to low levels. Similarly, when grouping all reported symptoms, individuals exposed to high levels of cyanobacteria were 1.7 (95%CI: 1.0–2.8) times more likely to report symptoms than their low-level cyanobacteria-exposed counterparts.

**Conclusion:**

A significant increase in reporting of minor self-limiting symptoms, particularly respiratory symptoms, was associated with exposure to higher levels of cyanobacteria of mixed genera. We suggest that exposure to cyanobacteria based on total cell surface area above 12 mm^2^/mL could result in increased incidence of symptoms. The potential for severe, life-threatening cyanobacteria-related illness is likely to be greater in recreational waters that have significant levels of cyanobacterial toxins, so future epidemiological investigations should be directed towards recreational exposure to cyanotoxins.

## Background

Planktonic cyanobacteria are common inhabitants of freshwater lakes and reservoirs throughout the world. Under favourable conditions, certain cyanobacteria can dominate the phytoplankton within a waterbody and form nuisance blooms. The principal public health concern regarding exposure to freshwater cyanobacteria relates to the understanding that some blooms produce toxins that specifically affect the liver or the central nervous system. Exposure routes for systemic poisoning by these toxins are oral, from accidental or deliberate ingestion of recreational water, and possibly by inhalation.

A small collection of case reports and anecdotal references dating from 1949 have described a range of illnesses associated with recreational exposure to cyanobacteria: hay fever-like symptoms, pruritic skin rashes and gastro-intestinal symptoms are most frequently reported. Some papers give convincing descriptions of allergic responses to cyanobacteria; others describe more serious acute illnesses, with symptoms such as severe headache, pneumonia, fever, myalgia, vertigo and blistering in the mouth. Anecdotal and case reports and the epidemiology of recreational exposure to freshwater cyanobacteria were recently reviewed by Stewart *et al *[1]. Under-reporting of minor, self-limiting illnesses may explain the small number of anecdotal and case reports in the literature. Moreover, a knowledge gap about cyanobacteria probably exists for many primary health care providers. Epidemiological studies into recreational exposure to cyanobacteria are also few in number. Five have been published to date: three cross-sectional studies from the United Kingdom using identical survey instruments [2-4], a small case-control analysis from Australia [5], and a larger prospective cohort study, also from Australia [6]. The UK studies and the smaller Australian study did not find any significant hazard from exposure to cyanobacterial blooms in recreational waters, but the study by Pilotto *et al *[6] reported an increase in illness amongst those exposed to relatively low levels of cyanobacteria (>5,000 cells per mL) compared to unexposed individuals.

Despite this limited and inconclusive evidence, the World Health Organization (WHO), Australia and several European countries have recommended guideline levels for recreational exposure to cyanobacteria [[7] (pp.149–54), [8]]. WHO guidelines present a three-tier approach, suggesting: 1) low probability of adverse health effects from waters with 20,000 cyanobacterial cells/mL or 10 μg chlorophyll-a/L (if cyanobacteria are dominant); 2) moderate probability of adverse effects from waters with 100,000 cells/mL or 50 μg chlorophyll-a/L; and 3) high probability of adverse effects from contact with and/or ingestion/aspiration of cyanobacteria at scum-forming densities [[7] (p.150)]. There is concern, however, that the current management practice in some countries (such as Australia or Germany) of warning all users or closing access to waterbodies is overly proscriptive. Such practices can result in unease amongst regular users of recreational waters that are affected by cyanobacteria, and can impact communities surrounding these waters, which are important social and economic resources.

Due to the small number of published epidemiology studies and the need for revision and refinement of recreational water exposure guidelines relating to planktonic cyanobacteria, we conducted a prospective cohort study to investigate morbidity following recreational exposure to cyanobacteria. Specifically, we sought to: 1) quantify cyanotoxins in designated water recreation sites, and 2) assess the relationship between exposure to cyanobacteria and cyanotoxins in recreational waters and the incidence of reported symptoms.

## Methods

The study population of interest comprised adults and children engaging in recreational activities in enclosed waters (i.e. not marine waters) inhabited to varying degrees by planktonic cyanobacteria. Subjects were recruited over a three-year period from 1999 to 2002 at water recreation sites in southern Queensland and the Myall Lakes area of New South Wales (Australia), and northeast and central Florida (USA). Recruitment was conducted on 54 separate days, mostly on weekends and holiday periods during the warmer months in order to maximise recruitment efficiency by concentrating on peak-use periods of recreational activity.

Entry criteria into the study were twofold:

• Engaging or planning to partake in water-contact activities in the study water body on the day of recruitment – ascertained by asking "Is anybody in the vehicle planning to go in the water and get wet here today?"

• Able to be contacted by phone for follow-up.

Study subjects were enrolled at the water sites and asked to complete a self-administered questionnaire before leaving for the day. They were also asked to submit to a telephone follow-up interview to be conducted as soon as practicable after three days from the day of enrolment. The interviewers asked to speak to study subjects within each household individually, i.e. proxy interviewees were discouraged. Exceptions were made in the case of children, where a parent or guardian was asked to decide whether or not their child would participate in the follow-up directly.

The questionnaire, follow-up interview form and information letter are available in Stewart [[9] (Appendix 1)]. The questionnaire gathered basic demographic data, information about relevant chronic conditions (chiefly allergies) and recent acute illnesses, as well as details of water-related activities. The follow-up interview elicited information about various acute illnesses, their onset and severity as well as smoking status, water exposure in the follow-up period and within-household grouping of study subjects.

Written permission (gatekeeper approval) from management authorities to recruit members of the public into this study was sought and secured for all sites listed in Table 1. The study was approved by the University of Queensland's Behavioural and Social Sciences Ethical Review Committee (clearance number B/168/SocPrevMed/99/PhD) and the University of Miami Human Subjects Committee (protocol number 02/031A).

**Figure 1:**
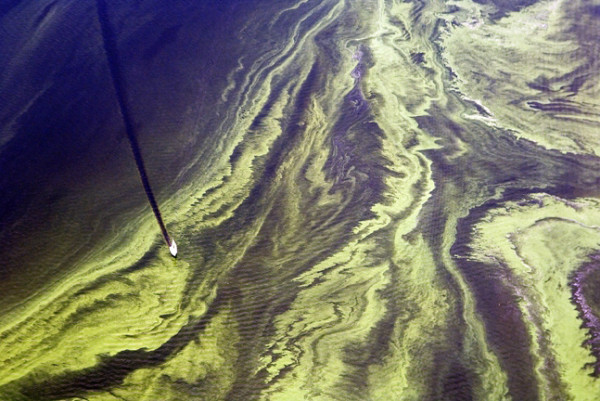
Aerial image showing a yacht traversing a *Microcystis *bloom, St Johns River, Florida, August 2005. Image copyright Bill Yates / CYPIX 2005.

Table 1 lists the main characteristics of the study sites. Some sites that were initially identified as potential study sites on the basis of cyanobacteria monitoring programs were subsequently found to have low cyanobacteria measures at the time of subject recruitment, and were thus classified as reference sites. Two sites served as both study and reference sites due to variability in cyanobacterial densities over several recruitment visits. Figure 1 is an aerial photograph of a recent cyanobacteria bloom affecting one of the study areas.

Water samples for phytoplankton and cyanotoxin analysis were collected by a modified grab sample method. Polypropylene sample bottles were used to collect water at a depth of approximately 70 cm; the modification involved moving the sample bottle up and down in a vertical plane to sample water through the entire column in order to avoid spurious cyanobacteria estimates through sampling only surface water. In an attempt to address temporal and spatial heterogeneity of cyanobacteria profiles within each waterbody, samples were collected from between one and four locations on each recruitment day, depending on the size of the site. Samples were collected in the morning and afternoon. All samples were kept on ice, in darkness, and equal volumes were then pooled prior to leaving the site to form a composite sample. Composite samples were immediately fixed with Lugol's iodine, then stored at 4°C until examined. Separate water samples were collected for cyanotoxin analysis; these samples were also stored at 4°C but were not fixed.

Sub-surface samples for faecal coliform analysis were collected in 250 mL sterile containers shortly before departing each site; containers were immediately placed on ice, and stored at 4°C until analysed. Due to logistical issues, faecal coliforms were sampled only when a recruitment visit was followed by a routine working day. Of the 54 study sampling days, coliform sampling was conducted on 21 days (i.e. 39% of exposure events included faecal coliform analysis).

Total phytoplankton analyses were conducted at three separate laboratories due to contractual obligations of the various agencies that funded this work: Queensland Health Scientific Services, Brisbane (National Association of Testing Authorities [NATA] accredited) for all Queensland samples; Australian Water Technologies, West Ryde, NSW (NATA accredited) for all Myall Lakes area samples; CyanoLab, Palatka, Florida for all Florida samples.

Cell identification and enumeration at these three centres were conducted by broadly similar methods, using a calibrated counting chamber with phase-contrast microscopy. Cell surface areas were determined by defining cyanobacteria cells as spherical or cylindrical, then measuring cell diameter (all cells) and length (idealised cylindrical cells). An appropriate number of cells were measured, and then averaged to give dimensions for each cyanobacterial taxon in each water sample. Cell surface areas were calculated using the formulas S.A. = 4*π*r^2 ^(idealised spherical cells), or S.A. = 2(*π*r^2^) + (2*π*r)l (idealised cylindrical cells) where v = cell volume; r = cell radius; l = cell length; S.A. = cell surface area. Data for each cyanobacterial taxon were summed, and total cyanobacterial cell surface area was used as the measure of exposure for each recruitment day in subsequent statistical analyses.

Samples that contained potentially toxic cyanobacteria were analysed for specific cyanotoxins:

• Microcystins : *Microcystis *spp, *Anabaena *spp, *Planktothrix *spp,

• Saxitoxins (Australia only): *Anabaena circinalis*

• Cylindrospermopsin: *Cylindrospermopsis raciborskii, Aphanizomenon ovalisporum*

• Anatoxin-a: (Florida only): *Anabaena *spp, *C. raciborskii*

Australian samples were analysed at Queensland Health Scientific Services laboratories. Saxitoxins were analysed by high performance liquid chromatography (HPLC) with fluorescence detection using a Shimadzu LC-10AVP system (Shimadzu Corp, Japan) based on the methods of Lawrence *et al *[10]; microcystins were measured by a Shimadzu LC-10A HPLC with photodiode array detection using the methods of Lawton *et al *[11]. Cylindrospermopsin was quantified by HPLC-MS/MS with a Perkin Elmer series 200 HPLC (Perkin Elmer Corp, USA) coupled to a PE SCIEX API 300 mass spectrometer (PE SCIEX, Canada) [12]. In Florida, toxins were analysed at CyanoLab. Cylindrospermopsin and anatoxin-a were determined by a HPLC-MS/MS method on a ThermoFinnigan LCQ Advantage system (ThermoFinnigan, USA). Microcystins were determined by an enzyme-linked immunosorbent assay method with a commercially available kit from Abraxis LLC (product # 520011, Abraxis LLC, USA).

All faecal coliform samples were analysed within 24 hours following collection. Samples were analysed at the following laboratories: Queensland Health Scientific Services, Brisbane, QLD (NATA accredited): method # AS 4276.7 (Australian Standard method for thermotolerant coliforms and *Escherichia coli *– membrane filtration method); Centre for Integrated Environmental Protection, Griffith University, Brisbane, QLD: method # APHA 9222D (APHA membrane filtration method); Forster Environmental Laboratory, Forster, NSW (NATA accredited): method # APHA 9222D; Columbia Analytical Services, Jacksonville, Florida (NELAC accredited): method # SM 9222D (USEPA Standard Method – membrane filtration method).

Water conductivity was measured at Australian study sites with an integrated conductivity/pH/temperature meter (Model WP-81, TPS P/L, Australia). In Florida, conductivity was recorded with a DataSonde MP 6600 (YSI Inc, USA).

## Data analysis

Cyanobacterial cell surface area was chosen as the principal exposure variable of interest [[9] (Chapter 3)] and classified as low (total cyanobacterial cell surface area <2.4 mm^2^/mL), intermediate (2.4–12.0 mm^2^/mL) and high (>12.0 mm^2^/mL) based on guidelines from the Queensland Department of Natural Resources and Mines [[9] (Chapter 3), [13]]. Faecal coliform exposures were categorised as positive or negative according to the Australian and New Zealand regulatory guidelines for fresh and marine water quality [14]. Participants were categorised into five age groups (see Table 2) and as active, passive or non-smokers. Passive smokers were defined as non-smokers or children aged less than 12 years who lived in a dwelling where at least one other household member smoked inside the house. Faecal coliform counts can fluctuate on a daily or weekly basis, so analyses including coliform data were conducted only on the sub-sets of the cohort for which these readings were available for the day of recruitment.

The dependent variable for all analyses was symptom reporting; symptoms were pooled into an "any symptom" category, and because of the disparate nature of symptoms associated with cyanobacteria exposure [1], reported symptoms were categorised as ear (sore ear/s; discharge from ear/s), eye (sore eye/s; eye redness; discharge from eye/s; itchy eye/s), gastro-intestinal (G-I) (vomiting; diarrhoea; abdominal pain; nausea), respiratory (difficulty breathing; dry cough; productive cough; runny nose; unusual sneezing; sore throat; wheezy breathing), cutaneous (skin rash; redness of the skin not related to sunburn; unusual itchiness), fever (single sign/symptom of fever) and the combined "any symptom" (any of the above). Respondents were asked to rate symptoms that occurred in the follow-up period as mild, moderate or severe. The number of symptoms reported as "severe" was, however, very low so this category was combined with "moderate", to form a single category. Subjects were excluded from specific analyses if they reported one or more associated acute symptoms had started before recruitment into the study.

Pearson's chi-squared test and Fisher's exact test were used to compare group proportions, while logistic regression was used to investigate associations between symptom variables and cyanobacterial exposure after accounting for potential confounding variables and geographic region, which was a design variable in all logistic regression models. A multivariable logistic regression main-effects model was developed, using sequential backward elimination of non-significant variables (based on the model deviance statistic). Once the most parsimonious main-effects model was identified, all two-factor interactions were introduced into the model and stepwise elimination of non-significant terms was undertaken (again based on the model deviance statistic) until the final model was obtained. The final model adjusted for age, sex, smoking and reported prior history of asthma, hay fever or eczema. A second multivariable model was developed for the "any symptoms" outcome by excluding subjects who reported exposure at the study waterbody in the five-day period prior to recruitment, as per the work of Pilotto *et al *[6]. SPSS v11.5 [15], Epi Info v6.O4d [16] and Stata/SE v8.0 [17] were used for statistical analyses and a significance level of α = 0.05 was used to define statistical significance.

## Results and discussion

The study entry criteria were met by 3,595 individuals; of these, 402 (11%) refused to participate in the study. Of the 3,193 people who accepted a questionnaire, 1,371 (43%) returned it. Of these, 40 individuals did not complete the follow-up interview for various reasons (uncontactable, refused). The 1,331 subjects with follow-up data thus represented 42% of those who initially accepted a questionnaire. Demographic features of the cohort are shown in Table 2. The majority of participants were from Queensland; most were less than 55 years of age and non-smokers.

Table 3 presents the proportion of subjects reporting symptoms at each level of cyanobacteria exposure. There were no significant differences between the frequency and reported severity of symptoms over the three cyanobacteria exposure groups. For further analyses, we collapsed the symptom variables into two groups of "not reported" and "reported at any severity". This dichotomisation also increased the robustness of the statistical modelling.

Table 4 presents the results of crude and multivariable logistic modelling. Two statistically significant findings were identified: compared to the low exposure group, reporting of both respiratory symptoms, odds ratio (OR) 2.1 (95%CI: 1.1–4.0), and the pooled "any symptom", OR 1.7 (95%CI: 1.0–2.9), was increased in the high exposure group. However, the significance of the latter result was not maintained with the exclusion of subjects with recent prior recreational water exposure, OR 1.6 (95%CI: 0.8–3.2).

Analysis of cyanotoxins in study waters showed that these were infrequently seen and, when seen, were at low levels. Microcystins were only detected on two occasions, at 1 μg/L (Doctors Lake) and 12 μg/L (Lake Coolmunda); cylindrospermopsin was found on seven occasions (Lakes Wivenhoe, Somerset, Atkinson and Seminole), but the levels were low at 1 μg/L and 2 μg/L. Saxitoxins were not seen in this study, and anatoxin-a was only detected at one Florida site (Lake Seminole) on a single recruitment day, at 1 μg/L. A statistically significant increase in symptom reporting amongst Florida subjects exposed to anatoxin-a was found by the Fisher-Freeman-Halton test (p = 0.04), but the number of subjects exposed (n = 18) was very low.

No relationship was seen between faecal coliform counts in study waters and symptom reporting: G-I symptoms (p = 0.50), respiratory symptoms (p = 0.92) and the pooled "any symptom" category (p = 0.96). Therefore we have no evidence that observed variation in symptom reporting could be attributed to differential exposure to enteric pathogens. However, our ability to monitor all recruitment days (mostly conducted on weekends and public holidays) for faecal coliforms was limited because the of the 24-hour maximum allowable time between sample collection and testing.

The main findings of this work were that individuals exposed to recreational waters from which total cyanobacterial cell surface areas exceeded 12 mm^2^/mL were more likely to report symptoms, particularly respiratory symptoms, after exposure than those exposed to waters where cyanobacterial cell surface areas were less than 2.4 mm^2^/mL. The measured effect size was similar but non-significant for ear and cutaneous symptoms, fever and all symptoms after exclusion of subjects with prior site exposure, which suggests that the sample sizes were too small to show significant differences within these categories. No relationship was detected between exposure to intermediate levels of cyanobacteria (total cell surface area 2.4–12.0 mm^2^/mL) and symptom reporting.

Although the symptom category that appeared to be weighting the pooled "any symptom" category was that of respiratory symptoms, from Table 3 we see that respiratory symptom reporting was skewed towards the "mild" symptom rating. Therefore, the conclusion that symptom reporting was higher in individuals exposed to high cyanobacteria levels must be tempered by the observation that most reported respiratory symptoms were mild.

This study attempted to improve on some study design weaknesses of previously published work in this field. The control group was recruited at waters known or suspected to be substantially free of cyanobacteria. We were concerned that the control subjects (i.e. non-bathers) in the studies of Pilotto *et al *[6] and Philipp [2], Philipp & Bates [3] and Philipp *et al *[4] might differ in some way from those who chose to go in the water. They might also tend to under-report relevant illnesses, given the propensity of some people to give the kind of answers that they think health researchers are seeking [18]. There is a risk that some individuals who are non-bathers, when presented with questions that are obviously concerned with water-related activities, might tend to downplay symptoms that they correctly assume are unrelated to water contact and then incorrectly assume to be of no interest to researchers. A control group of bathers also accounts for possible effects of water immersion that may be unrelated to water quality. Such effects may also lead to under-estimation of swimming-related illness when non-bathers comprise the unexposed comparison group [19].

We also measured cyanotoxins in study waters directly by HPLC-based methods. In previous studies cyanotoxins were either not considered or indirect and unquantified measures of cyanotoxin presence were used. However, the cyanotoxins were infrequently seen at study waters and, where seen, were at universally low levels. While we observed a significant increase in symptom reporting amongst Florida subjects exposed to anatoxin-a, the number of subjects exposed was very low, so we were reluctant to draw any conclusions from this finding. The infrequent presence and low concentrations of cyanotoxins in study waters highlights one of the disadvantages in conducting a prospective cohort study, that cyanobacteria and especially cyanotoxin levels are often dynamic and therefore unpredictable.

We chose a biomass estimate – cell surface area – to determine exposure to cyanobacteria, rather than the traditional reporting method of cell counts per unit volume of water [[9] (Chapter 3)]. Cyanobacteria cells can vary considerably in size, so measuring only cell counts will overestimate cyanobacterial biomass if picoplankton are dominant. Some workers recommend cyanobacterial biovolume or chlorophyll-a (if cyanobacteria dominate the phytoplankton profile) as estimates of cyanobacterial standing crop or for exposure guidelines [13,20].

We also analysed some study water samples for faecal indicator bacteria. Previous studies which did not incorporate such monitoring would have been unable to eliminate the potential contribution of enteric pathogens to specific symptom reporting.

The cohort was large enough to detect increased odds of symptom reporting in the "any symptom" and respiratory symptom categories amongst subjects exposed to high compared to low levels of cyanobacteria. Effects of similar magnitude were also seen for ear and skin symptoms and fever, as well as for symptom reporting after exclusion of subjects with recent prior exposure to study waters but these were not statistically significant. A larger sample size may have allowed us to confidently detect increased acute illness reporting from these symptom sub-groups. However, as this study essentially found only minor morbidity, the cost and effort required to conduct larger studies than this one would appear to outweigh the benefits.

We did not see any dose-response relationships. With the exception of a non-significant O.R. for febrile illness there was no increased reporting of symptoms at intermediate exposure but an increase at high exposure. One explanation for this may be a threshold effect, i.e. a minimum level of exposure is needed in order to see an effect.

**Figure 2:**
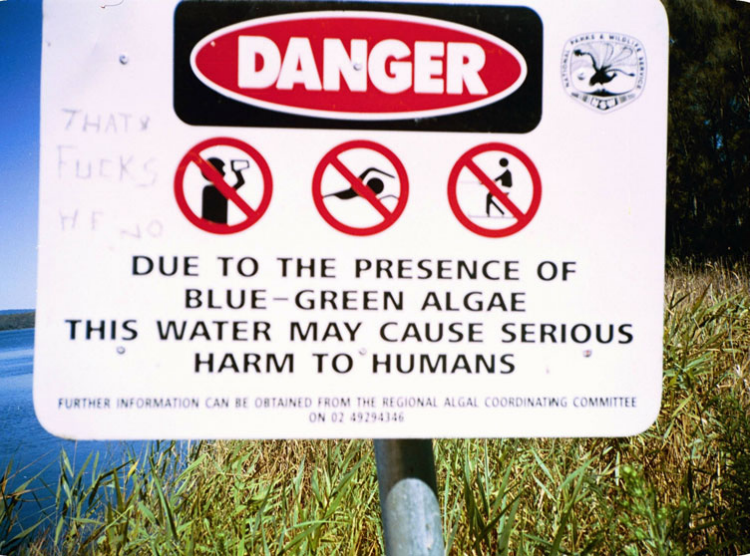
Example of an Australian cyanobacteria warning sign posted at a recreational water site (with a concise, eloquent but anonymous critique of the message?).

This work was a study of exposures to non-toxic and potentially toxic cyanobacteria in recreational waters, but with essentially little or no exposure to known cyanotoxins. Recommendations arising from this work cannot, therefore, be applied to situations where cyanobacterial exotoxins are found to be in possibly hazardous concentrations. Using levels of toxin-producing cyanobacteria as indirect measures of cyanotoxin presence may overestimate the public health risks; exposure guidelines and management strategies that address the potential, not actual, presence of hazardous levels of cyanotoxins run the risk of propagating "warning fatigue", where frequent or permanent advisories (see Figure 2) are ignored by a significant proportion of the public [[9] (Appendix 2), [21]].

A more rigorous assessment of the risks will come with regularly updated knowledge of the actual cyanotoxin content in recreational waters. At present, this knowledge is only obtained from testing of water samples in specialist cyanotoxin laboratories, which is expensive and with a lag period measured in days. Research strategies directed at an inexpensive, *in-situ *test for cyanotoxins would be appropriate. We suggest that future work in this field should investigate the epidemiology of recreational exposure to known cyanotoxins, rather than the epidemiology of recreational exposure to cyanobacteria *per se*.

When considering these results it is important to consider potential sources of error, particularly the possibilities of selection bias and confounding. Despite offering inducements (entry into a raffle for electronic goods, camping and boating permits) to increase participation in this study, the target population was inherently difficult to capture as most were healthy, young and busily engaged in leisure activities. The relatively low response rate (42%) means that the sample may become less representative of the wider population. The overall response rate also varied across the exposure groups with only 30% of eligible subjects returning questionnaires at high exposure sites compared to 43% and 44% of those at intermediate and low cyanobacteria sites respectively (p < 0.001). This difference was due to a particularly poor response from high exposure sites in Florida (27%). Some peculiar features of these sites in Florida probably contributed to the response rate, e.g. lack of swimming beaches (resulting in over-reliance on subjects using powered watercraft) and increased demand for limited parking spaces (manifested by boat-user etiquette for rapid site entry and egress, with subsequently reduced priority for completing questionnaires) [[9] (Appendix 2)]. However, assuming that those who failed to return questionnaires were no more or less likely to go on to develop symptoms than those who participated then, after adjusting for study region, the effect estimates should not be affected.

Because nuisance and potentially harmful cyanobacteria are cosmopolitan in distribution, and exposure guidelines should therefore be universal, not region specific, we combined the data from all three regions (Queensland, NSW and Florida). Overall, 80% of highly exposed subjects but only 10% of the low exposure group came from Florida. In addition, symptom reporting was considerably lower among Florida respondents than in Australia (OR for all symptoms = 0.6, 95%CI: 0.4–1.1 for Florida and 0.9, 95%CI: 0.6–1.3 for NSW compared to Queensland). Although we adjusted for region in our analyses, any residual confounding by this variable is likely to have weakened the true association. Of note, when we adjusted for important factors in our multivariable models, the symptom effect sizes associated with cyanobacteria exposure were strengthened slightly, suggesting that the associations seen are unlikely to be due to confounding. Although it is impossible to rule out other unknown confounders these would have to be strongly associated with both exposure and symptoms in order to completely explain the effects. We believe it unlikely that such strong confounders exist, nonetheless the possibility remains that unmeasured confounding variables may explain our findings.

## Conclusion

This study has shown that subjects exposed to high levels of cyanobacteria in recreational waters, as measured by total cell surface area, were more likely to report symptoms following such exposure than subjects exposed to low levels of cyanobacteria. Respiratory symptoms were most evident, and the reported severity of symptoms across all groups was low. Cyanotoxins, when detected in water samples, were present only at low concentrations throughout the course of the study. Further work quantifying the relationship between cyanotoxin levels and health outcomes should be considered. The potential remains for significant morbidity and possibly even mortality associated with recreational exposure to cyanotoxins, these being highly potent water-soluble toxins.

## Abbreviations

APHA American Public Health Association

G-I Gastro-intestinal

HPLC High performance liquid chromatography

HPLC-MS/MS HPLC + tandem mass spectrometry

NATA National Association of Testing Authorities, Australia

NELAC National Environmental Laboratory Accreditation Conference (USA)

OR Odds ratio

USEPA U.S. Environmental Protection Agency

WHO World Health Organization

## Competing interests

JWB was the director of CyanoLab and a former employee of St Johns River Water Management District at the time of field recruitment in Florida. No other authors have any competing interests.

## Authors' contributions

IS, PMW and GRS initiated the study conception and design. IS conducted field recruitment, water sample collection, data entry and manipulation and drafted the manuscript. IS and LEF conducted follow-up interviews. IS and PJS conducted statistical analyses. JWB, LEF, MG and LCB were involved in planning, logistics and site selection for recruitment of Florida subjects. GRS, PMW, PJS and LEF supervised the project. All authors participated in redrafting the manuscript. All authors read and approved the final manuscript.

## Pre-publication history

The pre-publication history for this paper can be accessed here:



## Figures and Tables

**Table 1:** Recreational waters visited for recruitment of study subjects

**SITE**	**REGION**	**DESCRIPTION**	**POWERED WATERCRAFT**	**PECULIAR FEATURES**	**(S)TUDY OR** **(R)EFERENCE SITE**	
					**PLANNED**	**ACTUAL**

Logan Inlet – Lake Wivenhoe	South-east Queensland	Large water-supply storage	No		S	S + R
Kirkleagh – Lake Somerset	South-east Queensland	Large water-supply storage	Yes		S	R
The Spit – Lake Somerset	South-east Queensland	Large water-supply storage	Yes		S	S + R
Brown Lake – Nth Stradbroke Island	South-east Queensland	Small dune lake	No	Acidic perched lake (pH 4.6 – 4.7)	R	R
Atkinson Dam	South-east Queensland	Small irrigation storage	Yes		S	S
Natural Arch	South-east Queensland	Upper catchment stream	No	Water temperature lowest of all sites	R	R
College's Crossing	South-east Queensland	Shallow river – release water from Wivenhoe Dam	No		R	R
Currumbin Rock Pool	South-east Queensland	Lower catchment stream	No	Dairy farms in catchment – faecal coliform counts often high	R	R
Bombah Broadwater	Myall Lakes NSW	Large saline enclosed lake	Yes		S	R
Myall Lake	Myall Lakes NSW	Large brackish water lake	No		S	S
Smith's Lake	Myall Lakes NSW	Small saline enclosed lake	Yes		R	R
Lake Coolmunda	Southern Queensland	Small irrigation storage	Yes		S	S
Hickory Point – Little Lake Harris	Central Florida	Chain of connected inland lakes	Yes		S	S
Manatee Springs	North/central Florida	Freshwater spring bulkhead	No		R	R
Fanning Springs	North/central Florida	Freshwater spring bulkhead	No		R	R
Lake Seminole	West/central Florida	Artificial lake in densely developed area	Yes		S	S
Doctors Lake	North-east Florida	Tidal influenced tributary lake of the St Johns River	Yes		S	S
Lake Cannon	Central Florida	Small urban lake	Yes		S	S
Lake Shipp	Central Florida	Small urban lake	Yes		S	S

**Table 2:** Demographic characteristics of the entire study cohort.

	***n***	**(%)**
***Age (years)***		
**<12**	312	(23)
**13–24**	346	(26)
**25–39**	375	(28)
**40–54**	256	(19)
**>55**	42	(3)
***Sex***		
**Female**	635	(48)
**Male**	696	(52)
***Region***		
**Florida**	216	(16)
**New South Wales**	300	(23)
**Queensland**	815	(61)
***Smoking status***		
**Non smoker**	953	(72)
**Passive smoker**	131	(10)
**Smoker**	247	(19)

**Table 3:** Frequency (percentage) of subjects reporting symptoms against level of cyanocyanobacteria exposure: low (cell surface area <2.4 mm^2^/mL), intermediate (2.4 – 12.0 m 12.0 mm^2^/mL) and high (>12.0 mm^2^/mL).

**Symptoms**		**Cyanobacteria exposure**			
		**Low *n*(%)**	**Intermediate *n*(%)**	**High *n*(%)**	**p-value**
***Any symptom***					0.42
	**None**	569 (70)	134 (74)	99 (67)	
	**Mild**	147 (18)	28 (16)	34 (23)	
	**Moderate/severe**	94 (12)	18 (10)	14 (10)	
***Ear symptoms***					0.75
	**None**	895 (96)	199 (98)	159 (96)	
	**Mild**	21 (2)	3 (2)	4 (2)	
	**Moderate/severe**	12 (1)	1 (1)	3 (2)	
***Eye symptoms***					0.75
	**None**	872 (94)	196 (95)	154 (95)	
	**Mild**	41 (4)	5 (2)	6 (4)	
	**Moderate/severe**	18 (2)	5 (2)	3 (2)	
***Gastro-intestinal symptoms***					0.89
	**None**	871 (94)	199 (95)	158 (95)	
	**Mild**	30 (3)	5 (2)	5 (3)	
	**Moderate/severe**	27 (3)	5 (2)	3 (2)	
***Respiratory symptoms***					0.57
	**None**	691 (86)	169 (88)	122 (82)	
	**Mild**	87 (11)	19 (10)	22 (15)	
	**Moderate/severe**	29 (4)	5 (3)	5 (3)	
***Cutaneous symptoms***					0.52
	**None**	875 (95)	197 (96)	159 (96)	
	**Mild**	23 (3)	6 (3)	6 (4)	
	**Moderate/severe**	20 (2)	2 (1)	1 (1)	

**Table 4:** Frequency (percentage) and binary logistic regression estimates of crude and multivariable O.R. and associated 95% confidence intervals (95%CI).

	**Exposure**	**Symptoms**			
	**Cyanobacterial cell surface area**^1^	**None**	**Present**	**Crude models**^2^	**Multivariable**^3^
		**n (%)**	**n (%)**	**O.R. (95%CI)**	**O.R. (95%CI)**
***Any symptom***					
	**Low**	569 (70)	241 (30)	1	1
	**Intermediate**	134 (74)	46 (26)	0.8 (0.6–1.2)	0.8 (0.6–1.3)
	**High**	99 (67)	48 (33)	**1.6 (1.0–2.7)**	**1.7 (1.0–2.9)**
***Ear symptoms***					
	**Low**	895 (96)	33 (4)	1	1
	**Intermediate**	199 (98)	4 (2)	0.6 (0.2–1.5)	0.5 (0.2–1.5)
	**High**	159 (96)	7 (4)	1.9 (0.6–6.5)	2.0 (0.6–7.1)
***Eye symptoms***					
	**Low**	872 (94)	59 (6)	1	1
	**Intermediate**	196 (95)	10 (5)	0.8 (0.4–1.8)	0.8 (0.4–1.9)
	**High**	154 (95)	9 (6)	0.6 (0.2–1.6)	0.6 (0.3–1.7)
***G-I symptoms***					
	**Low**	871 (94)	57 (6)	1	1
	**Intermediate**	199 (95)	10 (5)	0.9 (0.4–1.7)	0.9 (0.4–1.8)
	**High**	158 (95)	8 (5)	0.9 (0.3–2.2)	0.9 (0.4–2.2)
***Respiratory symptoms***					
	**Low**	691 (86)	116 (14)	1	1
	**Intermediate**	169 (88)	24 (12)	1.0 (0.6–1.6)	0.9 (0.6–1.6)
	**High**	122 (82)	27 (18)	**2.0 (1.1–3.7)**	**2.1 (1.1–4.0)**
***Cutaneous symptoms***					
	**Low**	875 (95)	43 (5)	1	1
	**Intermediate**	197 (96)	8 (4)	0.7 (0.3–1.7)	0.7 (0.3–1.8)
	**High**	159 (96)	7 (4)	1.9 (0.9–4.3)	1.9 (0.9–4.4)
***Fever***					
	**Low**	938 (99)	10 (1)	1	1
	**Intermediate**	208 (99)	3 (1)	1.5 (0.4–5.6)	1.8 (0.5–6.7)
	**High**	165 (99)	2 (1)	2.0 (0.5–8.7)	1.8 (0.4–8.1)
***Any symptom – after exclusion of subjects with recent (5 days prior to recruitment) exposure***					
	**Low**	436 (67)	200 (31)	1	1
	**Intermediate**	74 (69)	33 (31)	0.9 (0.6–1.5)	0.9 (0.6–1.5)
	**High**	71 (69)	32 (31)	1.5 (0.8–2.9)	1.6 (0.8–3.2)

^1^: Low = <2.4 mm^2^/mL; intermediate = 2.4 - 12.0 mm^2^/mL; high = >12.0 mm^2^/mL

^2^: Adjusted for geographic region (QLD, NSW, Florida) as a design variable and clustered by household

^3^: Adjusted for geographic region (QLD, NSW, Florida), age-group, sex, smoking and reported prior history of asthma, hay fever or eczema, and clustered by household
